# Wyburn-Mason disease: Management of a Spetzler-Martin grade 5 arteriovenous malformation with predominant thalamic involvement

**DOI:** 10.1016/j.radcr.2024.11.023

**Published:** 2024-12-12

**Authors:** Jovan Dhatt, Karis Houser, Kathryn A Szymanski, Kelly Halderman, Michael Kuwabara

**Affiliations:** a Midwestern University – Arizona College of Osteopathic Medicine, 19555 59^th^ Ave, Glendale, AZ 85308, USA; b Phoenix Children's Hospital, Department of Radiology, 1919 E Thomas Rd, Phoenix, AZ 85016, USA; c Creighton University, School of Medicine, 3100 N Central Ave, Phoenix, AZ 85012, USA

**Keywords:** Wyburn-Mason disease, Arteriovenous malformation, Trametinib, Thalamus, Spetzler-Martin Grade 5, Ischemia

## Abstract

Wyburn-Mason disease (WMD) is a rare congenital phakomatosis known for its complex arteriovenous malformations (AVMs) predominantly affecting the brain and ocular structures. We present the case of a 19-year-old female with an unruptured Spetzler-Martin grade 5 left thalamic AVM, who initially exhibited progressive visual impairment and migraines. Following diagnosis, she was treated with trametinib, a MEK inhibitor; however, nine months later, she developed acute complications, including left monocular blindness and right hemisensory loss. Imaging revealed narrowing of the left internal carotid artery and ischemia, leading to the discontinuation of trametinib. Her condition stabilized with gabapentin and supportive therapies. This case emphasizes the potential therapeutic role and risks of MEK inhibitors in managing high-grade AVMs in WMD and highlights the need for individualized management and careful monitoring in such cases.

## Introduction

Wyburn-Mason disease (WMD) is an uncommon, nonhereditary congenital phakomatosis characterized by arteriovenous malformations (AVMs) that commonly involve the central nervous system, ocular, and facial structures [1,2]. Brain AVMs in WMD frequently localize in critical regions such as the thalamus and hypothalamus, posing considerable clinical management challenges due to their high risk of hemorrhage and complex vascular anatomy [1]. These high-flow vascular lesions are associated with symptoms ranging from headaches and seizures to visual deficits, depending on lesion size and location [3,1,4]. Managing WMD-related AVMs, especially those of higher grades, remains challenging due to limited treatment options, as surgical resection is often high-risk, and conservative approaches may be insufficient.

Emerging pharmacologic treatments, such as MEK inhibitors like trametinib, have shown potential for controlling AVM progression in select cases. However, they carry risks that necessitate careful patient monitoring [5,6]. Here, we report a case of WMD with a high-grade thalamic AVM treated with trametinib. This case highlights both the therapeutic potential and complications associated with trametinib in managing WMD-associated AVMs, underscoring the need for an individualized approach to such complex vascular cases.

## Case presentation

The patient was a 19-year-old right-handed female with a past medical history of an unruptured Spetzler-Martin grade 5 left thalamic arteriovenous malformation (AVM). The patient experienced the onset of visual impairments eight years prior, which prompted periodic brain MR imaging. After 3 years without repeated imaging, the patient began experiencing migraines and deteriorating scholastic performance prompting repeat MRI. MRI showed progressive angioproliferative changes around the AVM nidus, and cerebral angiography of the AVM revealed multiple venous flow-related abnormalities. These findings allowed for diagnosis, and the patient was started on trametinib, a MEK inhibitor, due to its beneficial use in metameric spinal AVM patients. The patient presented nine months later with acutely worsening migraines, progressive left monocular blindness, and right hemisensory loss. MRI of the brain at this time reveals left internal carotid artery (ICA) narrowing and acute focal ischemia. Trametinib was discontinued for potential contributions to ischemic injury. Her deficits stabilized after completing occupational and physical therapy. The migraines improved with the ongoing regimen of gabapentin. The incidence of migraines with aura symptoms reduced to once every 3 months without incidence of debilitating migraines since the start of the medication. The patient reported well-controlled symptoms.

### Diagnosis

Imaging revealed ischemic injury related to a Spetzler-Martine grade 5 metameric, left diencephalic cerebral arteriovenous malformation expressing a Wyburn-Mason phenotype.

### Imaging findings

**Fig. 1:**
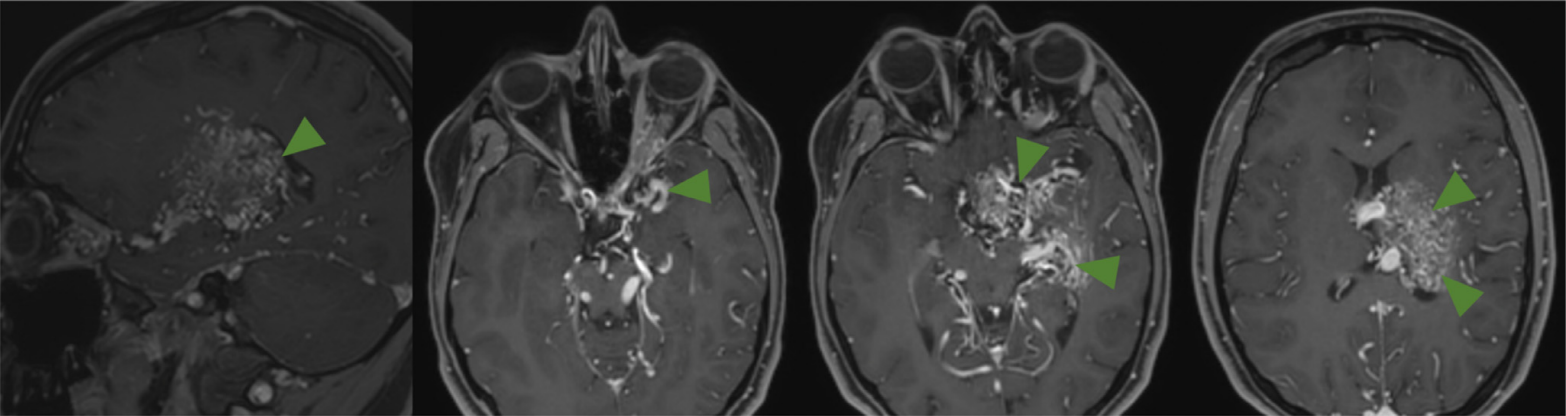
Postcontrast MRI brain at (A) sagittal and (B-D) axial cuts demonstrating the extent of the AVM (green arrows).

**Fig. 2:**
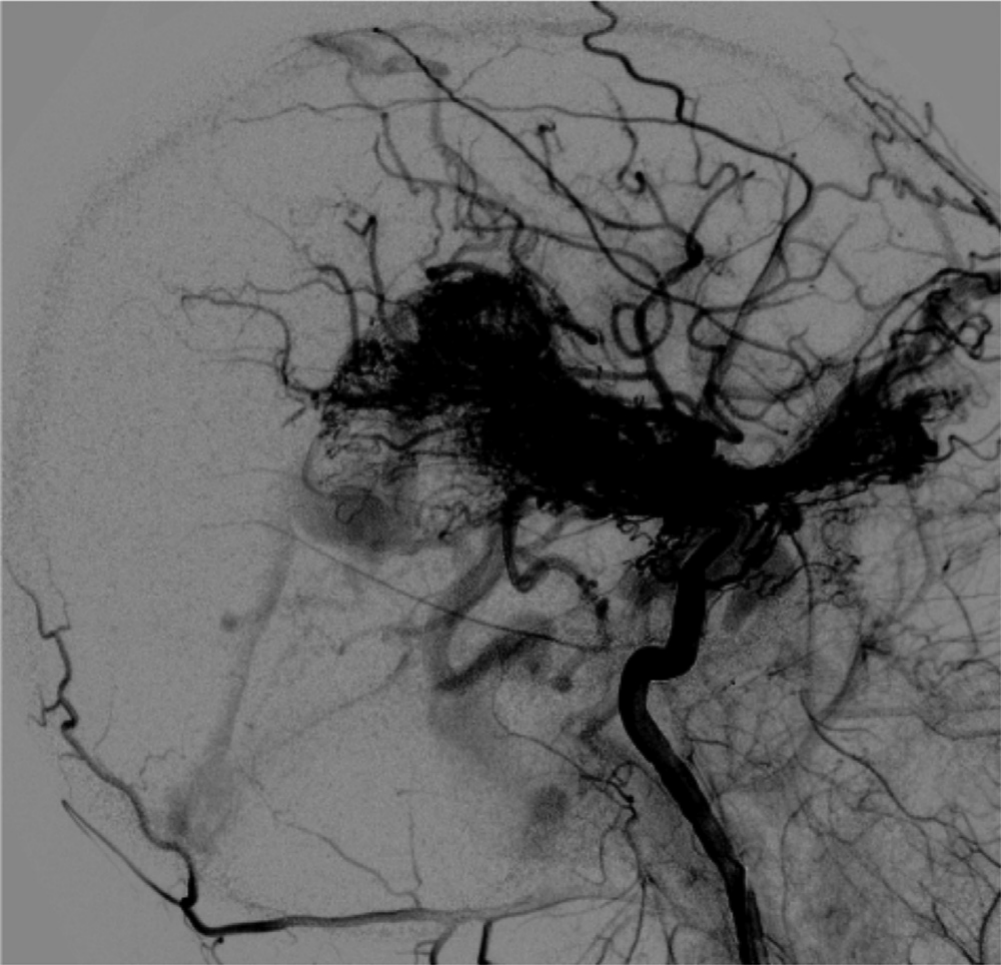
Conventional catheter-directed angiogram showing cranial AV malformation.

**Fig. 3:**
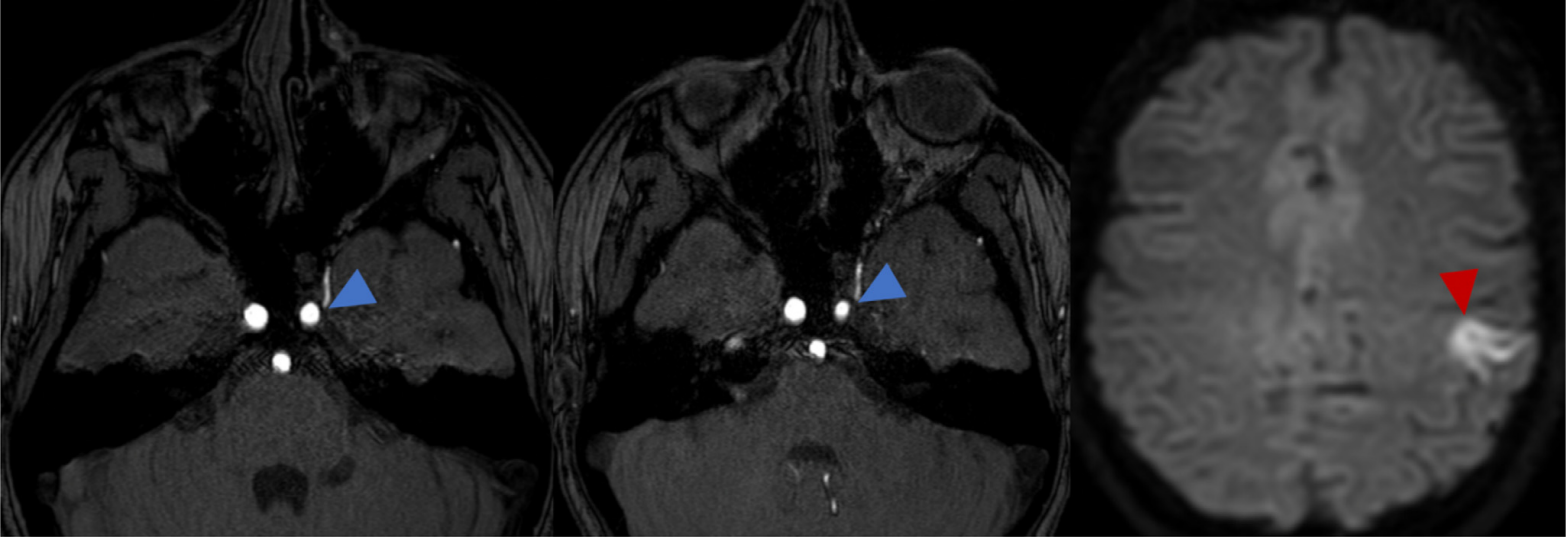
(A) Initial MRI brain before trametinib therapy with slightly small left ICA (blue arrow), and (B, C) MRI brain 9 months later after trametinib therapy with (B) progressive narrowing of left ICA (blue arrow) and (C) acute focal ischemia (red arrow).

At the time of presentation, MRI of the brain with contrast (Fig. 1) and cerebral angiography (Fig. 2) showed abnormal vasculature involving the left thalamus, globus pallidus, internal capsule, hypothalamus, suprasellar cistern, body of the left caudate nucleus, lateral putamen, and medial temporal tip as well as increased flow of different compartments of AVM in the left hemisphere compared to the right hemisphere. MRI of the brain 9 months after starting trametinib therapy showed a stable AV malformation. However, compared to the previous MRI, narrowing of the left internal carotid artery (ICA) and acute focal ischemic injury in the left postcentral gyrus were present (Fig. 3).

## Discussion

### Pathophysiology

Wyburn-Mason Disease (WMD) is a rare nonhereditary congenital phakomatosis that manifests sporadically. It is characterized by a complex array of vascular abnormalities affecting various organs, particularly the brain, eye, and facial structures [7,8]. The disease is distinguished by the presence of multiple AVMs that are notably large in size [1]. In the brain, these AVMs commonly localize in regions such as the hypothalamus, thalamus, optic chiasm, and midbrain. The precise etiology of Wyburn-Mason Disease remains unknown, though it is hypothesized to arise from embryonic defects resulting in the dissemination of vascular lesions [8]. Intracranial AVMs, characterized by high flow, pose significant clinical concerns due to the potential risk of hemorrhage.

### Clinical presentation and symptoms

The clinical presentation of WMD is intricately linked to the location and size of the AVM. Neurological manifestations are common with WMD affecting the brain and may include seizures, headaches, hemiparesis, visual deficits, cranial neuropathies, and hydrocephalus [[1], [3], [4], [9]]. The age at which WMD is diagnosed is contingent upon the presence of symptoms. Large or visually impairing AVMs prompt early diagnosis, while others may remain asymptomatic throughout the patient's lifespan [[8], [10]].

AVMs manifest on MRI as intricate clusters of vessels wherein arteries directly communicate with veins in the absence of a capillary bed. This presentation exhibits variability, spanning from a compact core of venous loops to a diffuse anomalous amalgamation of vessels dispersed within the brain parenchyma. Additionally, T1 and T2 weighted images discern the presence of rapid blood flow through enlarged arteries resulting in hallmark flow voids on imaging. AVM rupture presents with distinct stages of hemorrhage showcasing characteristic signal intensities on T1 and T2 [2]. A dark hemosiderin ring may be seen on T2 or gradient echo sequences. This is suggestive of remote hemorrhage [11].

In a comprehensive review conducted by Dayani and Sadun, an analysis of the anatomical distribution of intracranial AVMs across 27 published case reports was presented. The review revealed the most frequent locations for these AVMs, with the orbit being the most prevalent (17 out of 27 cases), followed by the hypothalamus/thalamus (11 out of 27), optic chiasm (7 out of 27), and suprasellar region (5 out of 27). Additionally, notable locations included the basal ganglia (4 out of 27), interpeduncular fossa (3 out of 27), and midbrain (3 out of 27) [1]. As orbit involvement accounts for the majority of literature discussion, here we highlight a less prominent but potentially catastrophic variation of WMD with a predominantly thalamic AVM.

### Diagnosis

Diagnosis of WMD brain malformations may involve magnetic resonance imaging (MRI), magnetic resonance angiography (MRA), computed tomography (CT), or cerebral angiography (CA). MRI is excellent at visualizing the AVM nidus and characterizing flow voids [12]. However, its utility can be limited in certain scenarios. In acute cerebral hemorrhage, the flow of compressed AVMs may become indiscernible on MRI, potentially leading to oversight, and necessitating serial MRI studies to uncover the underlying etiology. Additionally, MRI may underestimate the number of feeding arteries, potentially missing associated aneurysms [2]. Nonenhanced CT can identify small foci of calcification that are often associated with AVMs and may help delineate hyperattenuating serpiginous vessels that constitute the nidus [2]. Angiography, particularly digital subtraction angiography (DSA) may be used to confirm diagnosis of AVM and is indispensable for treatment planning and post-treatment monitoring. Offering the highest spatial and temporal resolution among neuroimaging modalities, angiography provides vital insights, including nidus configuration, vascular relationships, and drainage patterns of brain AVMs. DSA is essential for the identification of early draining veins without visible nidus, a critical risk factor for subsequent hemorrhage that is undetectable by other imaging modalities [13]. While angiography carries a low risk of immediate neurologic complications, particularly stroke, it uses large doses of radiation due to high frame rates and magnified views, potentially leading to long-term adverse effects [2].

### Treatment considerations

Treatment strategies for WMD are tailored to the precise location of AVMs and the associated clinical symptoms. In cases of unruptured AVMs, conservative management with close observation is often advocated. Treatment modalities for intracerebral AVMs encompass a spectrum of options, including radiation therapy, embolization, surgical resection, or a combined multidisciplinary approach tailored to individual patient needs [14]. Notably, interventions may carry significant risk which must be weighed with disease severity.

The Spetzler-Martin grading scale can be used to estimate surgical risk. Grades 4 and 5 lesions are considered to be at a high risk of surgical morbitity, and thus are typically treated with conservative medical management [15]. In the present case study described, the patient presented with a left thalamic AVM graded as Spetzler-Martin grade 4, prompting the adopting of supportive medical management.

Recent literature highlights the potential utility of MEK inhibitors in AVM management, attributed to their ability to reduce ERK activation and inhibit vascular network formation [5,6]. Consequently, this patient received trametinib therapy with the aim of impeding AVM progression. However, trametinib was stopped nine months after initiation due to clinical symptoms, including acutely worsening migraines, progressive left monocular blindness, and right hemisensory loss, and concerning changes seen on repeat brain MRI, including left ICA narrowing and acute focal ischemic injury in the left postcentral gyrus. While these findings may be unrelated, we report this to case to add to a growing body of research on intracranial AVM management.

### Outcomes

Intracranial AVMs harbor a reported rupture risk of approximately 1-3% per year, albeit with considerable variability depending on AVM characteristics [3]. For instance, while superficial venous drainage confers a lower risk of rupture (0.9%), deep-seated AVMs with intranidal aneurysms and deep venous drainage may pose a significantly elevated risk, reaching up to 34% [16]. Notably, the mortality risk associated with rupture is 29%, with 23% of survivors experiencing substantial long-term morbidity [3].

Ongoing discussions surrounding management render the assessment of prognosis challenging. As highlighted in the literature, the outcomes of the disease vary widely among affected individuals, complicating prognostic determination [3,4]. While some patients may remain asymptomatic throughout their lives, the course of the disease is often unpredictable and largely dependent on the management of associated symptoms. Complications of WMD affecting the brain can be diverse and may include intracranial hemorrhage or mechanical pressure-related damage [[1], [4], [7], [8], [17]].

## Patient consent

Complete written informed consent was obtained from the patient for the publication of this study and accompanying images.
